# Hybridization and Back-Crossing in Giant Petrels (*Macronectes giganteus* and *M*. *halli*) at Bird Island, South Georgia, and a Summary of Hybridization in Seabirds

**DOI:** 10.1371/journal.pone.0121688

**Published:** 2015-03-27

**Authors:** Ruth M. Brown, N. M. S. Mareile Techow, Andrew G. Wood, Richard A. Phillips

**Affiliations:** 1 Ecosystems Programme, British Antarctic Survey, Cambridge, United Kingdom; 2 Percy FitzPatrick Institute, DST/NRF Centre of Excellence, University of Cape Town, Cape Town, South Africa; Institute of Ecology, GERMANY

## Abstract

Hybridization in natural populations provides an opportunity to study the evolutionary processes that shape divergence and genetic isolation of species. The emergence of pre-mating barriers is often the precursor to complete reproductive isolation. However, in recently diverged species, pre-mating barriers may be incomplete, leading to hybridization between seemingly distinct taxa. Here we report results of a long-term study at Bird Island, South Georgia, of the extent of hybridization, mate fidelity, timing of breeding and breeding success in mixed and conspecific pairs of the sibling species, *Macronectes halli* (northern giant petrel) and *M*. *giganteus* (southern giant petrel). The proportion of mixed-species pairs varied annually from 0.4–2.4% (mean of 1.5%), and showed no linear trend with time. Mean laying date in mixed-species pairs tended to be later than in northern giant petrel, and always earlier than in southern giant petrel pairs, and their breeding success (15.6%) was lower than that of conspecific pairs. By comparison, mixed-species pairs at both Marion and Macquarie islands always failed before hatching. Histories of birds in mixed-species pairs at Bird Island were variable; some bred previously or subsequently with a conspecific partner, others subsequently with a different allospecific partner, and some mixed-species pairs remained together for multiple seasons. We also report the first verified back-crossing of a hybrid giant petrel with a female northern giant petrel. We discuss the potential causes and evolutionary consequences of hybridization and back-crossing in giant petrels and summarize the incidence of back-crossing in other seabird species.

## Introduction

Speciation, the process by which taxa evolve mechanisms conferring reproductive isolation, is generally considered to occur when geographically isolated populations gradually acquire genetic differences, either through selection or drift [1,2]. Reproductive isolation is achieved when genetic incompatibilities between sibling taxa become so great that offspring are infertile or unviable. If allopatric populations come into contact before this point is reached then there is the potential for interbreeding and genetic homogenization, unless behavioural isolating mechanisms, such as differences in the timing of breeding or incompatibility in mating displays, are sufficient to maintain reproductive isolation [3]. However, pre-copulatory barriers are often incomplete, leading to hybridization and gene flow between seemingly distinct taxa, particularly where divergence was relatively recent [4–6]. The study of hybridization in natural populations therefore provides important insights into the processes of evolutionary diversification, as well as contributing to accurate identification of species boundaries.

**Fig 1:**
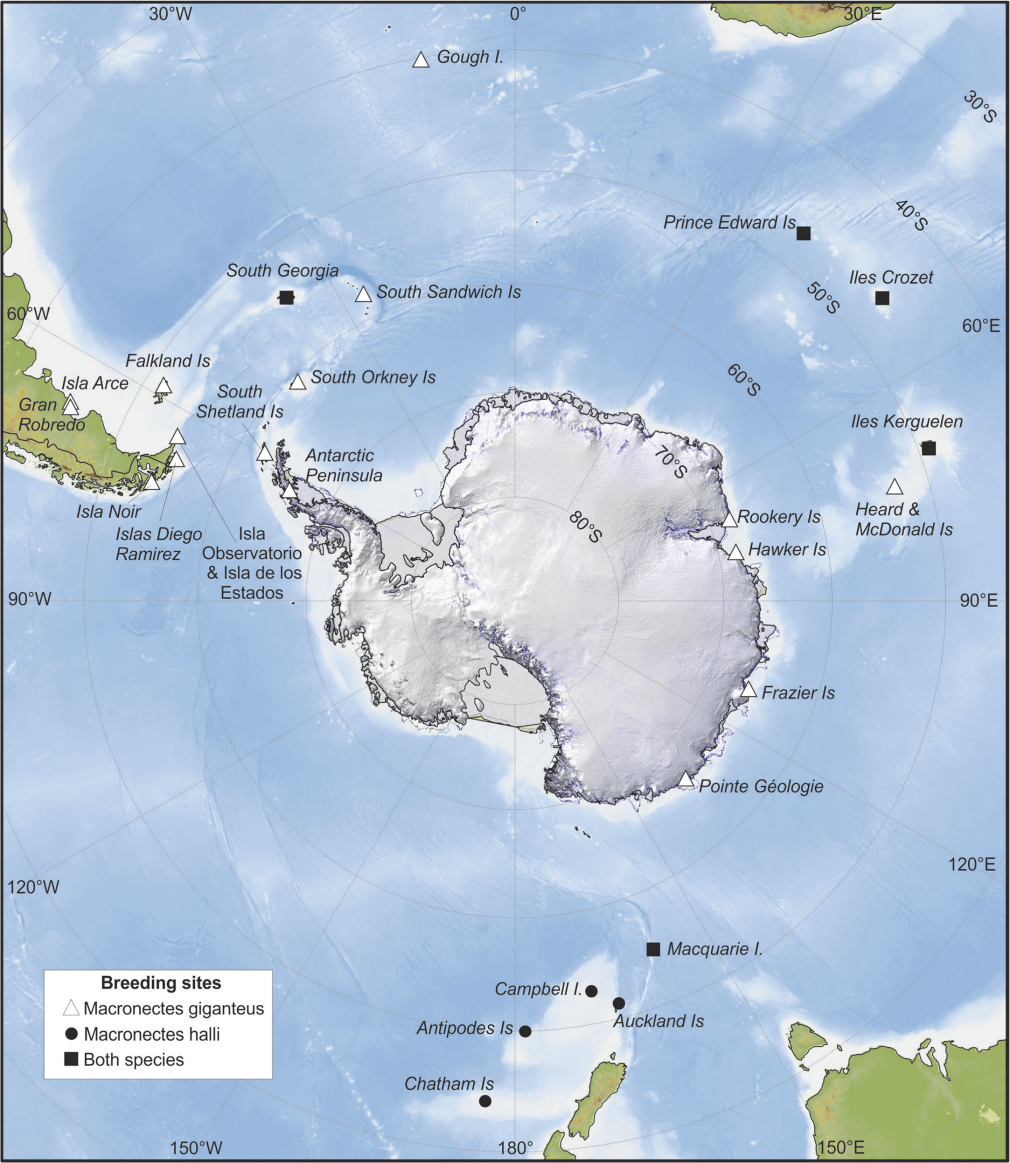
Breeding locations of northern (circles) and southern (triangles) giant petrels. Distribution data sourced from [9–11].

The two species of giant petrels are the only members of the genus *Macronectes*. Originally regarded as a single species, northern and southern giant petrels *M*. *halli* and *M*. *giganteus*, were spilt by Bourne & Warham [7] on the basis of morphological and behavioural differences, including the timing of breeding, colour of the bill tip and presence of a white morph in only one taxon (southern giant petrel). At some islands where the two species occur sympatrically there also appear to be differences in nest site selection [7,8]. Both species have a circumpolar breeding distribution; southern giant petrels breed both further north and further south than northern giant petrels, and are more widespread in the South Atlantic, whereas northern giant petrels are more common on islands around New Zealand (Fig. 1). The two species breed sympatrically at five island groups: South Georgia, the Prince Edward Islands, Îles Crozet, Îles Kerguelen and Macquarie Island (Fig. 1) [9–11], although only four breeding pairs of southern giant petrels have been recorded at Kerguelen, compared with around 1400 pairs of northern giant petrels [9]. The most recent estimates of global population sizes are 11,800 and 50,170 breeding pairs of northern and southern giant petrels, respectively [12,13].

Hybridization between northern and southern giant petrels has been reported at Marion Island, Macquarie Island and South Georgia (Bird Island). At Marion, unbanded mixed-species pairs were observed on eggs in the 1974/75 and 1976/77 breeding seasons, and a banded mixed-species pair (male southern giant petrel, female northern giant petrel) made at least eight breeding attempts between 1976/77 and 1995/96. Eggs were laid within the normal laying period for northern giant petrels but none hatched [14,15]. At Macquarie there is just one record of a breeding attempt by a mixed-species pair in the 1970/71 breeding season, which also failed before hatching [16]. At Bird Island six breeding attempts by mixed-species pairs (male southern giant petrel, female northern giant petrel) were recorded between 1978/79 and 1980/81, and all six chicks fledged successfully. In addition, nine breeding attempts were recorded between male southern giant petrels and putative hybrid females, and four chicks from these pairings fledged successfully [17]. These studies suggest that hybridization between northern and southern giant petrels is both more prevalent and more successful at Bird Island than elsewhere.

Systematic annual monitoring of ringed giant petrels of both species resumed at Bird Island in 2002/03. Using these data in combination with data collected between 1978/79 and 1980/81 by Hunter [17], we investigate various aspects of hybridization, including annual changes in the proportion of mixed-species pairs, timing of breeding, breeding success, and mate fidelity. We also report the first confirmed incidence of back-crossing between a hybrid and a northern giant petrel, summarise reports of hybridization in other seabird species, and discuss the potential causes and consequences of hybridization and back-crossing in these taxa.

## Materials and Methods

### Ethics statement

All animal work carried out during this study was conducted in accordance with UK Home Office guidelines and all bird ringing was undertaken by licensed ringers. The species sampled are not listed by CITES (Convention on the International Trade in Endangered Species). No permits were required for export of biological samples from South Georgia, and samples were imported into the UK under a DEFRA import licence (AHZ/2024A/2005/1). Fieldwork techniques involving live animals were approved by ethical review at the British Antarctic Survey.

### Annual monitoring

Chicks and adults of both northern and southern giant petrels were ringed by various field parties at Bird Island, South Georgia (54°00'S, 38°03'W) during the breeding season (Sept. to April) in 1958/59–1963/64 and 1972/73–1973/74 and a detailed study of breeding biology and population dynamics was carried out in 1978/79–1980/81 [17–19]. Systematic monitoring of ringed giant petrels resumed at Bird Island in the breeding season of 2002/03. During the laying period (late Sept.—early Dec.), a fixed study area of c. 28 hectares (c. 7% of the total area of the island) was checked at least weekly. All nests with an egg were marked with a wooden stake, the location recorded with a handheld GPS, and the nest visited weekly until the ring numbers of both incubating adults were recorded. Unringed adults were ringed during incubation in the first season in which they bred. All adults in the study area were identified to species based on the colour of the endplate (or unguis) of the bill, which is dark red in northern giant petrels, and pale green in southern giant petrels. Breeding adults were sexed by visual examination; males have a larger and deeper bill than females [20], and the accuracy of sexing checked by reference to the sex of the partner.

From 2002/03 to 2004/05, nests were visited 2–3 times during the hatching period to confirm successful hatching. Nests were visited again shortly before the fledging period, when the chicks were ringed and bill length recorded. From the start of the 2005/06 season, nests were visited every 1–2 days during laying in order to record laying dates, and then weekly thereafter to record dates of hatching and fledging, or failure date. Giant petrels usually breed annually and show high site and mate fidelity; therefore the data include breeding attempts by the same pairs in consecutive years. To avoid pseudo-replication comparisons of breeding success and laying dates were conducted separately for each year. All statistical analyses were conducted using Minitab v15.1 Statistical Software.

### Breeding of hybrid male and paternity testing

Since 2001/02, a number of breeding attempts by a ringed hybrid male (confirmed from the original ringing details) were recorded at Bird Island. DNA samples were collected from this hybrid, his long-term partner, and two of their chicks in the 2001/02 and 2004/05 breeding seasons. Likelihood of paternity was determined using analysis of microsatellite genotypes.

Total genomic DNA was extracted using the DNeasy Tissue Extraction Kit (Qiagen) following manufacturer’s instructions. Seven microsatellite loci were used for genotyping: Paequ3, Paequ4, De37, Dc16, Dc26, De11 and Dc5. Details of primers and PCR conditions are described in [10] (Paequ3, Paequ4, De37, Dc16, Dc26, De11) and [21] (Dc5). PCR products were electrophoresed on an ABI3730xl using POP7 and a 50cm capillary using Rox350 (Applied Biosystems) as the standard at the Central DNA Sequencing Facility of the University of Stellenbosch (http://www.sun.ac.za/saf). Profiles were analysed using GeneMapper Software version 3 (Applied Biosystems). CERVUS version 2.0 [22] was used to confirm paternity. The program uses a likelihood-based approach that allows for genotyping error and mutation, and by using simulations, can assign paternity at any statistical confidence. Thirty-two northern giant petrels blood-sampled at Bird Island for a previous genetic study [10] were used to calculate allele frequencies and to compute exclusion probabilities. A LOD (logarithm of odds) score was calculated for both mother, father and as a pair. LOD scores are the sum of the log-likelihood ratios at each locus calculated for each candidate. A LOD score of 3.0 means that the candidate father is highly likely to be the true father; whereas a score of -3.0 means that the candidate father is unlikely to be the true father. A LOD score between those two values is regarded as inconclusive [23].

## Results

### Incidence of mixed-species pairing

Three different types of mixed species pairs have been identified at Bird Island, referred to as Type A (male southern giant petrel x female northern giant petrel) [17], Type B (male southern giant petrel x hybrid female) [17] and Type C (hybrid male x female northern giant petrel) (this study). In Type B mixed-species pairs the female could not be identified to species based on bill colour, and so was assumed to be a hybrid [17]. Numbers of each type of mixed-species pair within the study area were recorded in 1978/79-1980/81 [17] and in ten recent breeding seasons 2002/03 to 2011/12 (Table 1). The proportion of all pairs which were mixed-species pairs (Types A, B and C) in the 13 years for which data are available, ranged from 0.37–2.41% (mean of 1.5%), and showed no significant trend over time (correlation, r = −0.018, p > 0.9).

**Table 1:** Numbers of conspecific and mixed-species pairs of giant petrels recorded at Bird Island, South Georgia, 1978/79 to 2011/12.

Breeding season	*M*. *halli*	*M*. *giganteus*	Total M. halli + M. giganteus	Type A southern ♂ northern ♀	Type B southern ♂ hybrid ♀	Type C hybrid ♂ northern ♀	Total mixed-species pairs	Proportion of mixed-species pairs (%)
1978/79	-	-	408	1	2	-	3	0.73
1979/80	-	-	284	3	4	-	7	2.41
1980/81	-	-	265	2	3	-	5	1.85
2002/03	156	111	267	1	-	-	1	0.37
2003/04	267	156	423	4	-	1	5	1.18
2004/05	261	155	416	5	-	1	6	1.44
2005/06	263	141	404	6	-	1	7	1.73
2006/07	280	150	430	2	-	1	3	0.70
2007/08	327	176	503	10	-	1	11	2.19
2008/09	363	185	548	11	-	1	12	2.19
2009/10	352	167	519	4	-	1	5	0.96
2010/11	296	131	427	8	-	1	9	2.11
2011/12	312	132	444	8	-	-	8	1.77

Type B mixed-species pairs—females were assumed to be hybrids based on bill colour [17].

Study area was different in 1978/79 to 1980/81.

### Breeding success

**Fig 2:**
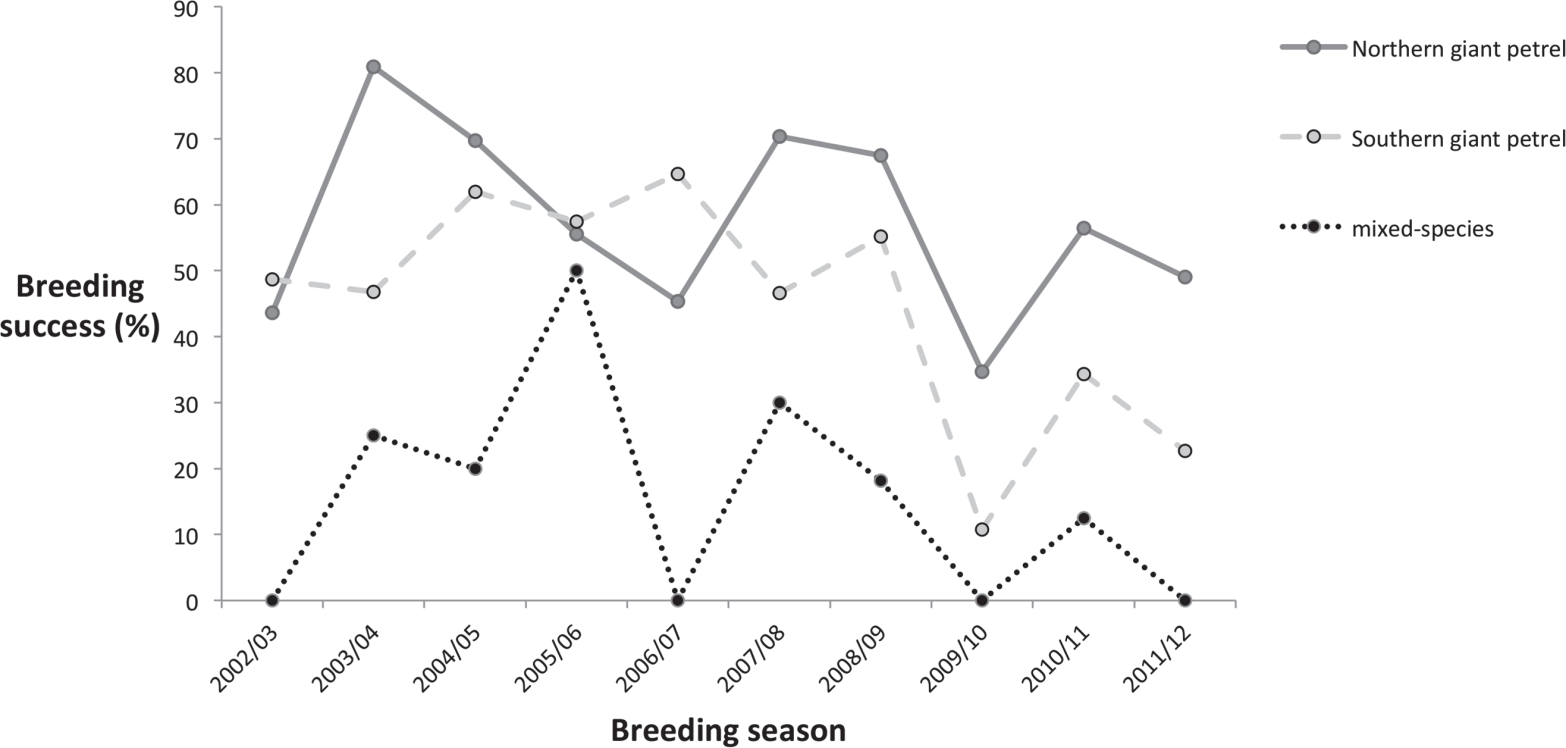
Breeding success (%) of giant petrels at Bird Island, South Georgia; 2002/03 to 2011/12.

There was considerable annual variation in breeding success (chicks fledged / eggs laid) of northern, southern and mixed-species pairs from 2002/03 to 2011/12 (Fig. 2). Average breeding success was 57.3% for northern giant petrels, 44.9% for southern giant petrels and 15.6% for Type A mixed-species pairs (male southern giant petrel x female northern giant petrel), although sample sizes for this last group each year are small. Breeding success of mixed-species pairs was significantly lower than that of conspecific pairs (Sign test, *n* = 10, p = 0.02). Of the eight breeding attempts by the Type C pair (hybrid male x female northern giant petrel) in 2002/03 to 2011/12, five chicks (62.5% of eggs) survived to fledging.

### Timing of breeding

**Fig 3:**
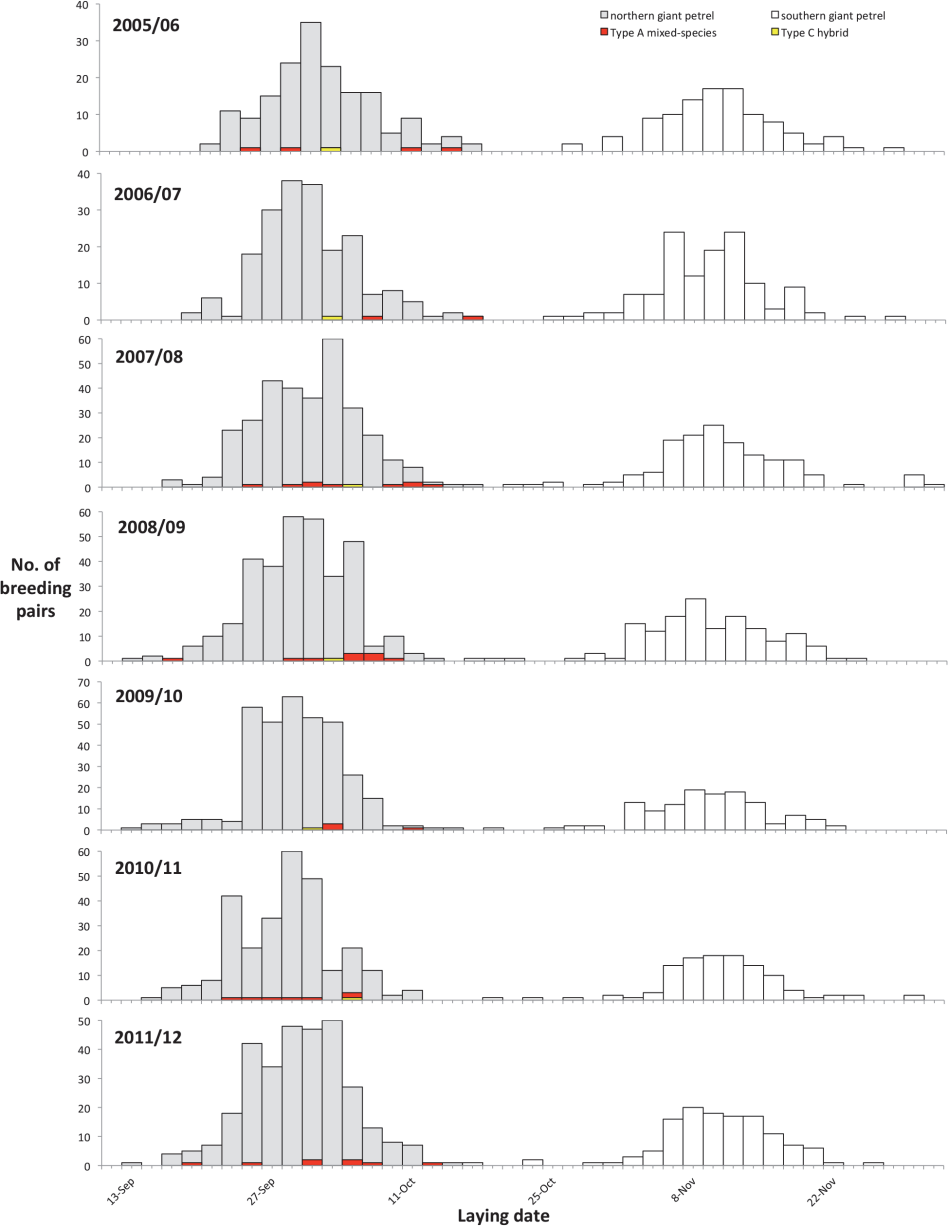
Distribution of laying dates for giant petrels at Bird Island, South Georgia; 2005/06 to 2011/12. Type A = southern ♂ x northern ♀; Type C = hybrid ♂ x northern ♀.

Laying dates of conspecific and Type A mixed-species pairs (male southern giant petrel x female northern giant petrel) were recorded between 2005/06 and 2011/12 (Table 2). Northern giant petrels lay around six weeks earlier than southern giant petrels, and the laying dates of Type A mixed species pairs showed much greater overlap with those of northern giant petrels (Table 2, Fig. 3). The mean laying date for Type A mixed-species pairs showed a tendency to be later than that of northern giant petrels pairs (Table 3), but mean laying date was not significantly different between the two groups in any season (p > 0.05). The laying date of the Type C mixed-species pair (hybrid male x female northern giant petrel) was on average on 4 October ± 1.7 days (range 1–6 October, *n* = 6) from 2005/06 to 2010/11, and overlapped with laying dates of northern giant petrels (Fig. 3).

**Table 2:** Laying dates (mean ± SD in days, sample size, and range in parentheses) of conspecific and Type A mixed-species pairs (southern ♂ x northern ♀) of giant petrels at Bird Island, South Georgia, 2005/06 to 2011/12.

Breeding season	*M*. *halli*	*M*. *giganteus*	southern ♂ northern ♀
2005/06	2 Oct ± 5.5, *n* = 173 **(**21 Sep—17 Oct)	12 Nov ± 5.8, *n* = 104 **(**28 Oct—29 Nov)	5 Oct ± 8.3, *n* = 5 **(**25 Sep—15 Oct)
2006/07	2 Oct ± 4.9, *n* = 198 (20 Sep—18 Oct)	11 Nov ± 5.5, *n* = 125 **(**26 Oct—29 Nov)	13 Oct ± 7.1, *n* = 2 **(**8 Oct—18 Oct)
2007/08	2 Oct ± 5.2, *n* = 313 (17 Sep—18 Oct)	12 Nov ± 6.8, *n* = 148 **(**22 Oct—2 Dec)	6 Oct ± 6.0, *n* = 10 **(**26 Sep—14 Oct)
2008/09	30 Sep ± 4.8, *n* = 330 (14 Sep—13 Oct)	10 Nov ± 6.3, *n* = 149 **(**18 Oct—24 Nov)	4 Oct ± 6.0, *n* = 11 **(**18 Sep—10 Oct)
2009/10	30 Sep ± 4.6, *n* = 325 (13 Sep—15 Oct)	10 Nov ± 5.9, *n* = 124 **(**19 Oct—23 Nov)	5 Oct ± 3.9, *n* = 4 **(**3 Oct—11 Oct)
2010/11	29 Sep ± 4.9, *n* = 262 (15 Sep—11 Oct)	11 Nov ± 6.3, *n* = 111 **(**20 Oct—1 Dec)	30 Sep ± 4.9, *n* = 8 **(**23 Sep—6 Oct)
2011/12	30 Sep ± 5.1, *n* = 312 (13 Sep—18 Oct)	11 Nov ± 5.9, *n* = 132 **(**17 Oct—27 Nov)	3 Oct ± 7.2, *n* = 8 **(**20 Sep—13 Oct)

**Table 3:** Results of t-tests (assuming unequal variance) comparing mean laying date of northern giant petrels and Type A mixed-species pairs (southern ♂ x northern ♀) in different years at Bird Island, South Georgia.

Breeding season	t	d.f.	p
2005/06	−0.69	4	0.53
2006/07	−2.25	1	0.27
2007/08	−2.20	9	0.06
2008/09	−1.89	10	0.09
2009/10	−2.77	3	0.07
2010/11	−0.80	7	0.45
2011/12	−0.85	7	0.43

### Mate fidelity

Fifty-nine breeding attempts by Type A mixed-species pairs (male southern giant petrel x female northern giant petrel) were recorded between 2002/03 and 2011/12. The majority of birds in mixed-species pairs were ringed (16 male southern giant petrels and 20 female northern giant petrels). In 10 recorded breeding attempts at least one partner was unringed, and in two of these breeding attempts both partners were unringed (young birds are sometimes too nervous to be ringed, but sex and species can be determined from a distance). Some birds in mixed-species pairs bred with the same partner in multiple years. Twelve pairs stayed together for two or more years and three of these pairs stayed together for at least five years. Twenty-two (9 males, 13 females) of the 36 ringed birds recorded in mixed-species pairs were only ever recorded in mixed-species pairs. Six birds (5 males, 1 female) switched partners during the study but remained in mixed-species pairs. Fourteen birds (7 males, 7 females) bred in conspecific pairs in one or more years as well as in mixed-species pairs. Of these 14 birds, eight (3 males, 5 females) were recorded in a mixed-species pair in just a single year, whereas six (4 males, 2 females) bred in mixed-species pairs in more than one year. The known hybrid male bred with the same female northern giant petrel in eight out of nine breeding attempts recorded between 2001/02 and 2010/11. In one season the nest failed before the identity of the hybrid’s partner was recorded.

### Breeding of hybrid male and paternity testing

The known hybrid male, his partner and two of their chicks were all successfully typed at seven microsatellite loci. Allelic mismatches were found between the first chick and its parents at locus De11 where it possessed an allele not found in either parent. The LOD scores for that chick were 6.02 (mother alone), 2.03 (father alone) and −1.36 (pair). For the second chick LOD scores were 3.79 (mother alone), 2.94 (father alone) and 4.41 (pair). The second chick was assigned at 95% confidence and no mismatches were observed for both parents. The first chick was assigned at 80% confidence levels but given its negative LOD score, paternity is inconclusive. However, a single mismatch may be explained by mutation or genotyping error (in this case the mismatch was confirmed by re-amplifying DNA from the chick and from both parents) and therefore in many studies a single mismatch is tolerated [23].

## Discussion

This study provides the first evidence that hybrid offspring of northern and southern giant petrels are fertile and that back-crossing occurs. The known hybrid male, ringed as a chick, was observed breeding in nine seasons and successfully raised five chicks with the same female northern giant petrel. Genetic analysis indicated that he was highly likely to be the father of at least one of the two chicks that were sampled. Therefore it is probable that some level of gene flow between northern and southern giant petrels occurs at Bird Island. Based on the ratio of mixed-species to conspecific pairs from 1978/79 to the present, the rate of hybridization has remained more or less constant for around 30 years. However, given that the population size of northern giant petrels has increased over that time [24], the absolute number of mixed-species pairs on the island has probably also risen.

Inter-specific hybridization is relatively common in birds; around 9% of species are known to form hybrid pairs in nature, and since some hybridization events will be rare or cryptic, the true frequency is likely much higher [25]. The incidence of hybridization varies considerably among the orders of birds, and appears to be more frequent in terrestrial birds than in seabirds [25,26]. Nevertheless, multiple examples of hybridization exist in four of the five orders that include seabirds (Procellariiformes, Sphenisciformes, Pelecaniformes and Charadriiformes; (S1 Table)). In most cases hybridization in seabirds has been inferred by the observation of mixed-species pairs engaging in courtship behaviour or incubating eggs or chicks, or by the presence of individuals that are morphologically intermediate between two putative parent species (S1 Table), although in more recent studies genetic data have been useful in documenting hybridization [27]. In some instances putative hybrids have been observed breeding, but back-crossing of hybrids with one or both parental species has been confirmed in only four studies including our own (although strongly suspected in three additional cases) (S1 Table).

### Causes of hybridization

Hybridization generally occurs following range expansion and secondary contact between taxa that have not yet evolved complete reproductive isolation, and most avian hybrid zones in the Northern Hemisphere appear to be the result of post-Pleistocene range expansions [28]. The two species of giant petrels most likely evolved in isolation when an ancestral giant petrel population became fragmented due to climatic changes, with subsequent range expansion leading to secondary contact in some regions [10] and consequent hybridization.

Hybridization in birds is often unidirectional; hybrid pairs form between males of species A and females of species B but not vice versa [5,29,30]. This appears to be the case for giant petrels, where all allospecific pairs recorded thus far have involved a male southern giant petrel and a female northern giant petrel. Female choice is thought to be an important component of unidirectional hybridization, potentially influenced by factors such as unequal sex ratios in colonies, sex-biased dispersal, female preference for allospecific males, and the greater rarity of one species [29]. These scenarios are thought to explain some occurrences of unidirectional hybridization in seabirds, eg. [5,29,30–32]; however it is unclear which, if any, explain unidirectional hybridization in giant petrels. The sex ratio of the adult northern giant petrel population on Bird Island is unknown, therefore this cannot be ruled out as a possible influence on hybridization. Dispersal patterns in these species are also unknown. There are no obvious differences in sexual display or plumage between the two species that might act as supernormal stimuli, therefore it is unlikely that female northern giant petrels preferentially mate with male southern giant petrels. Wirtz [29] concluded that where one species is rare, hybrid pairs will form between females of the rare species and males of the common species. The opposite pattern is seen on Bird Island, where northern giant petrels are more numerous. However, Randler [33] disputed that females in hybrid pairs will always be the rare species, citing several opposing examples in birds and concluding that hybridization is simply encouraged by restricted mate choice.

One further alternative explanation for unidirectional hybridization in giant petrels at Bird Island is based on allochrony. At all sites where they breed sympatrically, northern giant petrels lay around six weeks earlier than southern giant petrels [34]. However, breeding of both species is several weeks later at South Georgia than at other breeding sites, such that the laying dates of northern giant petrels at South Georgia overlap with those of southern giant petrels at colonies in the Indian Ocean, Macquarie and Gough islands. Hence, an immigrant male southern giant petrel from one of those populations that arrived at South Georgia in breeding condition would encounter reproductively active female northern, but not southern giant petrels. Those females might be more likely to accept allospecific males because their regular partner had failed to return and most other male conspecifics were already breeding, which would tie in with the trend towards later breeding of mixed-species pairs at Bird Island recorded in our study.

### Consequences of hybridization

Hybridization in natural populations can have a variety of evolutionary consequences: reinforcement of reproductive isolation, the formation of a new species, fusion or genetic swamping of one species, the formation of a stable hybrid zone, or transfer of genetic material between species [35]. The outcome of a given hybridization event depends on the fertility of hybrid offspring, their fitness relative to parental species and the environmental conditions under which hybridization is occurring. Examples of reinforcement, and the formation of new species, resulting from hybridization have been recorded in the literature [36–38]; however such outcomes are considered to be rare [39]. Genetic swamping tends to occur when one species is uncommon [40] or when introduced species hybridise readily with native species [41]. Stable hybrid zones form when hybridization is restricted to a narrow zone of contact between the parental species, but the genetic integrity of both parental species remains intact [35]. Hybrid zones have been identified in the wild in a variety of taxa, and have proved extremely useful in the study of the hybridization process [42–45]. Perhaps the most significant evolutionary consequence of hybridization is the transfer of genetic material between species, which potentially facilitates the rapid creation of novel genotypes and can lead to adaptive evolution [26,33,46]. Even if hybridization is limited to a narrow hybrid zone, positive selection can drive introgressed DNA beyond this zone and lead to replacement sweeps across entire species [46]. This seems to occur more frequently with mitochondrial DNA, perhaps because its role in basic metabolic function has important consequences for individual fitness, particularly across different temperature regimes [46]. However, there is a potential barrier to transmission of mtDNA in birds known as ‘Haldane’s rule’, which predicts that heterogametic hybrid offspring are often less viable or fertile than homogametic hybrid offspring [47]. Several hypotheses have been advanced for the genetic cause of Haldane’s rule [48,49]. One possibility is that recessive sex-linked genes that reduce hybrid fitness are more likely to be expressed in the heterogametic sex, which carry only a single copy of each allosome. In the homogametic sex there is the possibility for recessive sex-linked genes to be masked by a corresponding dominant gene [46]. In birds, females are the heterogametic sex, and Haldane’s rule has been shown to hold true in 51 out of 53 bird species examined [50]. Haldane’s rule implies that introgression of mtDNA, which is transmitted maternally, is unlikely in hybridizing bird species, since a female F1 hybrid is required to introduce mtDNA into the recipient species. Nevertheless, evidence of introgressed mtDNA has been discovered in most bird families [46].

In seabirds there are at least two examples of supposed replacement of mtDNA as a result of ancient hybridization events, in the pomarine skua *Stercorarius pomarinus* [51] and in the Rapa shearwater *Pufinus myrtae* [52]. That mtDNA selective sweeps occur in birds despite Haldane’s rule reveals that female hybrid offspring are viable and fertile in some cases. Even if female F1 hybrids are rare, there is potential for large scale introgression of DNA through back-crossing. Ferris *et al* [53] suggested that a single individual *Mus domesticus* hybridizing with Scandinavian *M*. *musculus* resulted in replacement of *musculus* mtDNA with *domesticus* mtDNA in their study populations. Thus hybridization, even involving very few individuals, can have a profound and lasting effect on the genotypes of the species involved.

The evolutionary significance of hybridization between giant petrel species at Bird Island remains to be determined. Globally, the majority of northern and southern giant petrel populations breed in allopatry, and on the five island groups where they breed sympatrically, successful hybridization has only been reported at South Georgia. Breeding success of mixed-species pairs is lower than that of conspecific pairs, and to date only a single known hybrid has been recorded breeding. Genetic swamping of one species by the other seems highly unlikely, and it is possible that South Georgia represents a stable hybrid zone for these species. Our results demonstrate that back-crossing does occur in giant petrels, and therefore there is potential for the transfer of genetic material between species. A recent genetic analysis involving samples from the whole breeding range provided no evidence of introgression [10]. However, a more detailed genetic analysis at South Georgia, the only location where hybridization is known to be successful, may be more revealing. The transfer of mtDNA between species would require the pairing of a female F1 hybrid with a male southern giant petrel. At least one female hybrid chick has survived to fledging in recent years (RMB pers. obs.), and the long-term population monitoring on Bird Island makes the detection of such a pairing more likely, should it occur, leading to the tantalizing possibility of observing genetic introgression via hybridization in real time.

### Implications for species status of giant petrels

Northern and southern giant petrels fulfil the criteria for species status proposed by the Taxonomic Sub-committee of the British Ornithological Union (BOU) [54], despite evidence that hybridization and gene flow are occurring in natural populations. The morphological and behavioural differences between the two giant petrel taxa (colour of bill tip, presence of white morph in only one species, six week difference in mean laying date), make them easily distinguishable in the field. With regard to hybridization, the BOU allows taxa to be ranked as species ‘if they hybridize only rarely, so that gene flow occurs at such low frequency that it is unlikely their gene pools will ever merge’ [54]. Given that numerous populations of both northern and southern giant petrels breed in allopatry, that successful hybridization appears to be restricted to South Georgia and occurs at a low level, and that a recent large scale study of the population structure of giant petrels found consistent genetic differences between the two taxa [10], it is improbable that genetic fusion of the two species will occur. Therefore, in agreement with Techow *et al* [10] and the taxonomic assessment of giant petrels by ACAP [55] we see no reason to change the species status for northern and southern giant petrels.

## Supporting Information

S1 Table Examples of hybridization (species A x species B) in seabirds. EPC = extra-pair copulation, JUV = juvenile.
(DOCX)

## References

[1] Dobzhansky T (1940) Speciation as a stage in evolutionary divergence. Am Nat 74: 312–321.

[2] Mayr E (1942) Systematics and the origin of species from the viewpoint of a zoologist Columbia University Press, New York.

[3] Harrison RG (1993) Hybrid zones and the evolutionary process Oxford University Press, Oxford, UK.

[4] Taylor SA, Anderson DJ, Zavalaga CB, Friesen VL (2012) Evidence for strong assortative mating, limited gene flow, and strong differentiation across the blue-footed/ Peruvian booby hybrid zone in northern Peru. J Avian Biol 43: 311–324.

[5] Taylor SA, Patirana A, Birt T, Friesen V (2012) Cryptic introgression between murre sister species (*Uria* spp.) in the Pacific low Arctic: frequency, cause and implications. Polar Biol 35: 931–940.

[6] Brown RM, Nichols RA, Faulkes CG, Jones CG, Bugoni L, Tatayah V, et al (2010) Range expansion and hybridization in Round Island petrels (*Pterodroma* spp.): evidence from microsatellite genotypes. Mol Ecol 19: 3157–3170. 10.1111/j.1365-294X.2010.04719.x 20618891

[7] Bourne WRP, Warham J (1966) Geographical variation in the giant petrels of the genus *Macronectes* . Ardea 54: 45–67.

[8] Hunter S (1987) Species and sexual isolating mechanisms in sibling species of giant petrels *Macronectes* . Polar Biol 7: 295–301. 3429607

[9] Patterson DL, Woehler EJ, Croxall JP, Cooper J, Poncet S, Peter H-U, et al (2008) Breeding distribution and population status of the Northern Giant Petrel *Macronectes halli* and the Southern Giant Petrel *M*. *giganteus* . Mar Ornith 36: 115–124.

[10] Techow NMSM, O’Ryan C, Phillips RA, Gales R, Marin M, Patterson-Fraser D, et al (2010) Speciation and phylogeography of giant petrels *Macronectes* . Mol Phyl Evol 54: 472–487. 10.1016/j.ympev.2009.09.005 19755164

[11] Brooke M (2004) Albatrosses and Petrels Across the World. Oxford University Press, Oxford.

[12] Agreement on the Conservation of Albatrosses and Petrels (2010) ACAP Species Assessment: Northern Giant Petrel *Macronectes halli* Downloaded from http://www.acap.aq, 9 Dec 2014.

[13] Agreement on the Conservation of Albatrosses and Petrels (2010) ACAP Species Assessment: Southern Giant Petrel *Macronectes giganteus* Downloaded from http://www.acap.aq, 9 Dec 2014.

[14] Burger AE (1978) Interspecific breeding attempts by *Macronectes giganteus* and *M*. *halli* . Emu 78: 234–235.

[15] Cooper J, Brooke M de L, Burger AE, Crawford RJM, Hunter S, Williams TAJ (2001) Aspects of the breeding biology of the Northern Giant Petrel (*Macronectes halli*) and the Southern Giant Petrel (*M*. *giganteus*) at sub-Antarctic Marion Island. Int J Ornith 4: 53–68.

[16] Johnstone GW (1978) Interbreeding by *Macronectes halli* and *M*. *giganteus* at Macquarie Island. Emu 78: 235.

[17] Hunter S (1983) Interspecific breeding in Giant Petrels at South Georgia. Emu 82 (supplement): 312–314.

[18] Hunter S (1983) The food and feeding ecology of the giant petrels *Macronectes halli* and *M*. *giganteus* at South Georgia. J Zool London 200: 521–538.

[19] Hunter S (1984) Breeding biology and population dynamics of giant petrels *Macronectes* at South Georgia (Aves: Procellariiformes). J Zool London 203: 441–460.

[20] Copello S, Quintana F, Somoza G (2006) Sex determination and sexual size-dimorphism in Southern Giant petrels (*Macronectes giganteus*) from Patagonia, Argentina. Emu 106: 141–146.

[21] Burg T (1999) Isolation and characterisation of microsatellites in albatrosses. Mol Ecol 8: 335–346. 10065551

[22] Marshall TC, Slate J, Kruuk LEB, Pemberton JM (1998) Statistical confidence for likelihood-based paternity inference in natural populations. Mol Ecol 7: 639–655. 9633105 10.1046/j.1365-294x.1998.00374.x

[23] Slate J, Marshall T, Pemberton J (2000) A retrospective assessment of the accuracy of the paternity inference program CERVUS. Mol Ecol 9: 801–808. 10849296 10.1046/j.1365-294x.2000.00930.x

[24] González-Solís J, Croxall JP, Wood AG (2000) Foraging partitioning between giant petrels *Macronectes* spp. and its relationship with breeding population changes at Bird Island, South Georgia. Mar Ecol-Prog Ser 204: 279–288.

[25] Grant PR, Grant R (1992) Hybridization of bird species. Science 256: 193–197. 17744718 10.1126/science.256.5054.193

[26] Arnold ML (1997) Natural Hybridization and Evolution. Oxford University Press, New York, USA.

[27] Taylor SA & Friesen VL (2012) Use of molecular genetics for understanding seabird evolution, ecology and conservation. Mar Ecol Prog Ser 451: 285–304.

[28] Price T (2008) Speciation in birds Roberts and Company, Colorado, USA.

[29] Wirtz P (1999) Mother species-father species: unidirectional hybridization in animals with female choice. Anim Behav 58: 1–12. 10413535 10.1006/anbe.1999.1144

[30] Taylor SA, Zavalaga CB, Friesen VL (2010) Hybridization between blue-footed (*Sula nebouxii*) and Peruvian (*Sula variegata*) boobies in Northern Peru. Waterbirds 33: 251–257.

[31] Moore PJ, Burg TM, Taylor GA, Millar CD (2001) Provenance and sex ratio of Black-browed Albatross, *Thalassarche melanophrys*, breeding on Campbell Island, New Zealand. Emu 101: 329–334.

[32] Robertson CJR (1993) Timing of egg laying in the Royal Albatross (*Diomedea epomophora*) at Taiaroa Head 1937–1992. Conservation Advisory Science Notes No. 50, Dept. Of Conservation, Wellington, New Zealand.

[33] Randler C (2002) Avian hybridization, mixed pairing and female choice. Anim Behav 63: 103–119.

[34] Marchant S, Higgins PJ (1990) Handbook of Australian, New Zealand and Antarctic Birds. Vol 1: Ratites to Ducks Oxford University Press, Melbourne.

[35] Seehausen O (2004) Hybridization and adaptive radiation. Trends in Ecol Evol 19: 198–207. 16701254 10.1016/j.tree.2004.01.003

[36] Sternkopf V, Liebers-Helbig D, Ritz MS, Zhang J, Helbig AJ, de Knijff P (2010) Introgressive hybridization and the evolutionary history of the herring gull complex revealed by mitochondrial and nuclear DNA. BMC Evol Biol 10: 348 10.1186/1471-2148-10-348 21067625 PMC2993719

[37] Smith PF, Konings A, Kornfield I (2003) Hybrid origin of a cichlid population in lake Malawi: implications for genetic variation and species diversity. Mol Ecol 12: 2497–2504. 12919487 10.1046/j.1365-294x.2003.01905.x

[38] Hermansen JS, Sæther SA, Elgvin TO, Borge T, Hjelle E, Sætre G-P (2011) Hybrid speciation in sparrows I: Phenotypic intermediacy, genetic admixture and barriers to gene flow. Mol Ecol 20: 3812–3822. 10.1111/j.1365-294X.2011.05183.x 21771138

[39] Barton NH (2013) Does hybridization influence speciation? J Evol Biol 26: 267–269. 10.1111/jeb.12015 23324003

[40] Bertellotti M, Donázar JA, Blanco G, Forero G (2003) Imminent extinction of the guanay cormorant on the Atlantic South American coast: a conservation concern? Biodivers Conserv 12: 743–747.

[41] Huxel GR (1999) Rapid displacement of native species by invasive species: effects of hybridization. Biol Cons 89(2): 143–152.

[42] Concannon MR, Stein AC, UY JAC (2012) Kin selection may contribute to lek evolution and trait introgression across an avian hybrid zone. Mol Ecol 21: 1477–1486. 10.1111/j.1365-294X.2012.05474.x 22320709

[43] Carling MD, Zuckerberg B (2011) Spatio-temporal changes in the genetic structure of the *Passerina* bunting hybrid zone. Mol Ecol 20: 1166–1175. 10.1111/j.1365-294X.2010.04987.x 21232074

[44] Maroja LS, Andres JA, Harrison RG (2009) Genealogical discordance and patterns of introgression and selection across a cricket hybrid zone. Evol 63: 2999–3015. 10.1111/j.1558-5646.2009.00767.x 19619226

[45] Gay L, Crochet PA, Bell DA, Lenormand T (2008) Comparing clines on molecular and phenotypic traits in hybrid zones: a window on tension zone models. Evol 62: 2789–2806. 10.1111/j.1558-5646.2008.00491.x 18752618

[46] Rheindt FE, Edwards SV (2011) Genetic introgression: an integral but neglected component of speciation in birds. Auk 128: 620–632.

[47] Haldane JSB (1922) Sex-ratio and unisexual hybrid sterility in animals. J Genet 12: 101–109.

[48] Orr HA (1993) Haldane's rule has multiple genetic causes. Nature 361: 532–533. 8429905 10.1038/361532a0

[49] Wu CI, Davis AW (1993) Evolution of postmating reproductive isolation: the composite nature of Haldane's rule and its genetic bases. Am Nat 142 (22): 187–212. 19425975 10.1086/285534

[50] Orr HA (1997) Haldane’s Rule. Ann Rev Ecol Syst 28: 195–218.

[51] Andersson M (1999) Hybridization and skua phylogeny. Proc R Soc London B 266: 1579–1585.

[52] Austin JJ, Bretagnolle V, Pasquet E (2004) A global molecular phylogeny of the small *Puffinus* shearwaters and implications for systematics of the little-Audubon’s shearwater complex. Auk 121: 847–864.

[53] Ferris SD, Sage RD, Huang CM, Nielsen JT, Ritte U, Wilson AC (1983) Flow of mitochondrial DNA across a species boundary. Proc Natl Acad Sci USA 80: 2290–2294. 6300907 10.1073/pnas.80.8.2290 PMC393805

[54] Helbig AJ, Knox AK, Parkin DT, Sangster G, Collinson M (2002) Guidelines for assigning species rank. Ibis 144: 518–525.

[55] Agreement on the Conservation of Albatrosses and Petrels (2010) Taxonomic Working Group—Report. Downloaded from http://www.acap.aq, 15 October 2012.

