# Supplementary material for: Hybridization and Back-Crossing in Giant Petrels (Macronectes giganteus and M. halli) at Bird Island, South Georgia, and a Summary of Hybridization in Seabirds
Source: PLoS One. 2015 Mar 27;10(3):e0121688. doi: 10.1371/journal.pone.0121688 (PMC4376808; doi:10.1371/journal.pone.0121688)
Supplement: S1 Table — EPC = extra-pair copulation, JUV = juvenile. (DOCX) [file pone.0121688.s001.docx]

|  |  | | |  |  |  |  |  |  |  |  |  |
| --- | --- | --- | --- | --- | --- | --- | --- | --- | --- | --- | --- | --- |
| **Species A** | |  | **Species B** |  | **Location / years** | **Hybrid adult (putative)** | **Hybrid adult (known)** | **Mixed-species pair** | **Mixed-species pair with egg or chick** | **Hybrid adult breeding** | **Back-crossing confirmed** | **References** |
| **Procellariiformes** | |  |  |  |  |  |  |  |  |  |  |  |
| Northern giant petrel | | *Macronectes halli* | Southern giant petrel | *M. giganteus* | S Georgia 1978 – 2012, Marion Is. 1974 – 1995, Macquarie Is. 1970 | + | + | + | + | + | + | This study, 1, 2, 3, 4 |
| Kermadec petrel | | *Pterodroma neglecta* | Trindade petrel | *P. arminjoniana* | Round Is., Mauritius 2004 - 2005 | + |  | + | + | + | + | 5, 6 |
| Campbell albatross | | *Thalassarche impavida* | Black-browed albatross | *T. melanophrys* | Campbell Is, NZ |  | + | + | + | + |  | 7, 8, 9 |
| Northern royal albatross | | *Diomedea sanfordi* | Southern royal albatross | *D. epomophora* | Taiaroa Head, NZ 1962 - 1969 |  | + | + | + | + |  | 10 |
| Laysan albatross | | *Phoebastria immutabilis* | Black-footed albatross | *P. nigripes* | NW Hawaiian Is. late 1800s - 2010 | + |  |  |  | + |  | 11, 12, 13, 14, RB pers. obs. |
| Yelkouan shearwater | | *Puffinus yelkouan* | Balearic shearwater | *P. mauretanicus* | Menorca ~2007 |  |  | + |  |  | suspected | 15, 16 |
| Newell’s shearwater | | *Puffinus newelli* | Little shearwater (Rapa Is. subsp.) | *P. assimilis myrtae* | ancient |  |  |  |  |  | suspected | 17 |
| Short-tailed shearwater | | *Puffinus tenuirostris* | Sooty shearwater | *P. griseus* |  | + |  |  |  |  |  | 18 |
| **Sphenisciformes** | |  |  |  |  |  |  |  |  |  |  |  |
| Humboldt penguin | | *Spheniscus humboldti* | Magellanic penguin | *S. magellanicus* | Puñihuil and Metalqui Is., S. Chile ~2009 | + | + | + | + | + |  | 19 |
| Rockhopper penguin | | *Eudyptes chrysocome* | Macaroni penguin | *E. chrysolophus* | Falkland Is. 1998 – 2001, Heard & Marion Is. 1987 | + |  | + | + | + |  | 20, 21 |
| Rockhopper penguin | | *Eudyptes chrysocome* | Royal penguin | *E. schlegeli* | Macquarie Is. 1957, Campbell & Macquarie Is. ~1999 | + |  | + |  |  |  | 22, 23 |
| Erect-crested penguin | | *Eudyptes sclateri* | Royal penguin | *E. schlegeli* | Macquarie Is. 1964 |  |  | + |  |  |  | 23 |
| Rockhopper penguin | | *Eudyptes chrysocome* | Erect-crested penguin | *E. sclateri* | Falkland Is. ~1968, Campbell Is. ~1999 |  |  | + |  |  |  | 22, 24 |
| **Pelecaniformes** | |  |  |  |  |  |  |  |  |  |  |  |
| Blue-footed booby | | *Sula nebouxii* | Peruvian booby | *S. variegata* | Lobos de Tierra & Lobos de Afuera Is., Peru 1997 - 2007 | + | + | + |  | + | suspected | 25, 26, 27, 28 |
| Masked booby | | *Sula dactylatra* | Brown booby | *S. leucogaster* |  | + |  |  |  |  |  | 29 |
| Masked booby | | *Sula dactylatra* | Nazca booby | *S. granti* | Clipperton & San Benedicto Is. | + |  |  |  |  |  | 30 |
| Blue-footed booby | | *Sula nebouxii* | Nazca booby | *S. granti* | Lobos de Tierra Is. 1999 |  |  | + |  |  |  | 27 |
| Lesser frigatebird | | *Fregata ariel* | Great frigatebird | *F. minor* | Tern Island, Hawaii 1998 | + |  | + EPC |  | + |  | 31 |
| King cormorant | | *Phalacrocorax albiventer* | Guanay cormorant | *P. bougainvilli* | Chubut Province, Argentina 1980 - 1999 | + |  | + | + | + |  | 33 |
| King cormorant | | *Phalacrocorax albiventer* | Imperial shag | *P. atriceps* | Chubut Province, Argentina 1980 - 1985 | + JUV |  |  |  |  |  | 33 |
| **Charadriiformes** | |  |  |  |  |  |  |  |  |  |  |  |
| Common murre | | *Uria aalge* | Thick-billed murre | *U. lomvia* | Norway ~1968 & ~1993, Newfoundland 1980, North Pacific | + |  |  | + | + | + | 34, 35, 36, 37 |
| Common murre | | *Uria aalge* | Razorbill | *Alca torda* | Newfoundland 1996 – 2002 | + |  |  |  |  |  | 38 |
| Roseate tern | | *Sterna dougallii* | Arctic tern | *S. paradisaea* | Nova Scotia, Canada 1998 |  |  | + | + |  |  | 39 |
| Roseate tern | | *Sterna dougallii* | Common tern | *S. hirundo* | Connecticut & Great Gull Is., New York, 1972 | + |  | + | + | + |  | 40, 41, 42 |
| Black tern | | *Chlidonias niger* | White-winged black tern | *C. leucopterus* |  | + JUV |  |  |  |  |  | 43, 44 |
| Gull-billed tern | | *Gelochelidon nilotica* | Foster’s tern | *Sterna fosteri* |  |  |  |  |  |  |  | 45 |
| Lesser crested tern | | *Thalasseus bengalensis* | Sandwich tern | *T. sandvicensis* | Valencia 1998 | + JUV |  | + |  |  |  | 46, 47, 48, 49 |
| Sandwich tern | | *Thalasseus sandvicensis* | Elegant tern | *T. elegans* | California 1995 |  |  | + |  |  |  | 50 |
| Chilean skua | | *Stercorarius chilensis* | South Polar skua | *S. maccormicki* | King George Island, Antarctica 1993 - 1995 | + |  |  |  |  |  | 51 |
| South Polar skua | | *Stercorarius maccormicki* | Brown skua | *S. skua lonnbergi* | Antarctic Peninsula, South Orkney & South Shetland Is. |  |  |  |  |  |  | 52, 53, 54 |
| Falkland skua | | *Stercorarius skua antarctica* | Chilean skua | *S. chilensis* | Southern Argentina |  |  |  |  |  |  | 55 |
| Pomarine skua | | *Stercorarius pomarinus* | Great skua | *S. skua* | ancient |  |  |  |  |  | suspected | 56 |
| Great black-backed gull | | *Larus marinus* | American herring gull | *L. smithsonianus* | ancient |  |  |  |  |  | suspected | 57 |
| Great black-backed gull | | *Larus marinus* | Herring gull | *L. argentatus* | NW Atlantic | + |  |  |  |  |  | 58, 59, 60, 61 |
| Herring gull | | *Larus argentatus* | Glaucous gull | *L. hyperboreus* | Iceland 1964 – 2006, Mackenzie Delta, Canada 1984 | + | + | + | + | + | suspected | 63, 64, 65, 66 |
| Glaucous-winged gull | | *Larus glaucescens* | Western gull | *L. occidentalis* | Pacific coast, N. America 1908 - 1990 | + |  | + |  | + |  | 67, 68 |
| Herring gull | | *Larus argentatus* | Lesser black-backed gull | *L. fuscus* | France 1983 – 1990, UK |  |  | + | + |  |  | 69, 70 |
| Lesser black-backed gull | | *Larus fuscus* | Caspian gull | *L. cachinnans* | France 1983 - 1990 |  |  | + | + |  |  | 69 |
| Herring gull | | *Larus argentatus* | Caspian gull | *L. cachinnans* | Russia & Poland 1998 - 2004 | + |  | + |  | + | + | 71, 72 |
| Glaucous-winged gull | | *Larus glaucescens* | Herring gull | *L. argentatus* | Alaska 1961 – 1962 | + |  |  |  |  |  | 73 |
| Glaucous-winged gull | | *Larus glaucescens* | Glaucous gull | *L. hyperboreus* | Alaska 1972 – 1974 | + |  |  |  |  |  | 74 |
| Glaucous-winged gull | | *Larus glaucescens* | Slaty-backed Gull | *L. schistasagus* | Kamchatka Peninsula |  |  |  |  |  |  | 75 |
| Black-billed gull | | *Chroicocephalus bulleri* | Red-billed gull | *C. scopulinus* | Rotorua, New Zealand 1964 – 1965 | + |  | + | + | + |  | 76 |
| Brown-headed gull | | *Chroicocephalus bruunicephalus* | Black-headed gull | *C. ridibundus* | Central Asia |  |  |  |  |  |  | 76 |
| Black-headed gull | | *Chroicocephalus ridibundus* | Mediterranean gull | *Ichthyaetus melanocephalus* | Mediterranean, UK 1968 |  |  |  |  |  |  | 77, 78 |
| Hartlaub’s gull | | *Chroicocephalus hartlaubii* | Grey-headed gull | *C. cirrocephalus* | South Africa 1977 |  |  | + |  |  |  | 79 |

**Table S1. Examples of hybdridization (species A x species B) in seabirds. EPC = extra-pair copulation, JUV = juvenile.**

**References**

1. Techow, NMSM, O’Ryan, C, Phillips, RA, Gales, R, Marin, M, Patterson-Fraser, D, Quintana, F, Ritz, MS, Thompson, DR, Wanless, RM, Weimerskirch, H and Ryan, PG (2010) Speciation and phylogeography of giant petrels *Macronectes*. Mol Phyl Evol 54: 472-487.
2. Cooper, J, Brooke, M de L., Burger, AE, Crawford, RJM, Hunter, S & Williams, TAJ (2001) Aspects of the breeding biology of the Northern Giant Petrel (*Macronectes halli*) and the Southern Giant Petrel (*M. giganteus*) at sub-Antarctic Marion Island. Int J Ornith 4: 53-68.
3. Burger, AE (1978) Interspecific breeding attempts by *Macronectes giganteus* and *M. halli*. Emu 78: 234-235.
4. Johnstone, GW (1978) Interbreeding by Macronectes halli and M. giganteus at Macquarie Island. Emu 78: 235.
5. Brown, RM, Nichols, RA, Faulkes, CG, Jones, CG, Bugoni, L, Tatayah, V, Gottelli, D & Jordan, WC (2010) Range expansion and hybridization in Round Island petrels (*Pterodroma* spp.); evidence from microsatellite genotypes. Mol Ecol 19: 3157-3170.
6. Brown, RM, Jordan, WC, Faulkes, CG, Jones, CG, Bugoni, L, Tatayah, V, Palma, RL & Nichols, RA (2011) Phylogenetic relationships in *Pterodroma* petrels are obscured by recent secondary contact and hybridization. *PloS ONE* 6(5): e20350.
7. Burg, TM & Croxall, JP (2001) Global relationshipsamongst black-browed and grey-headed albatrosses: analysis of population structure using mitochindrial DNA and microsatellites. Mol Ecol 10: 2647-2660.
8. Moore, PJ, Taylor, GA & Amey, JM (1997) Interbreeding of black-browed albatross *Diomedea m. melanophrys* and New Zealand black-browed albatross *D. m .impavida* on Campbell Island. Emu 97: 322-324. In [9].
9. Moore, PJ, Burg, TM, Taylor, GA & Millar, CD (2001) Provenance and sex ratio of Black-browed Albatross, *Thalassarche melanophrys*, breeding on Campbell Island, New Zealand. Emu 101: 329-334.
10. Robertson, CJR (1993) Timing of egg laying in the Royal Albatross (*Diomedea epomophora*) at Taiaroa Head 1937-1992. Conservation Advisory Science Notes No. 50, Dept. Of Conservation, Wellington, New Zealand.
11. Rothschild, W (1900) The avifauna of Laysan and the neighboring islands, with a complete history to date of the Hawaiian possessions*.* Porter Ltd, London, UK. In [14].
12. Fisher, HI (1948) Interbreeding of Laysan and black-footed albatrosses**.** Pacific Science 2: 132. In [14].
13. Fisher, HI (1972) Sympatry of Laysan and black-footed albatross. Auk 89: 381-402. In [14].
14. McKee, T & Pyle, P (2002) Plumage variation and hybridization in black-footed and Laysan albatrosses. N Amer Birds 56: 131-138.
15. Genovart, M, Oro, D, Juste, J & Bertorelle, G (2007) What genetics tell us about the conservation of the critically endangered Balearic shearwater? Biol Cons 137: 283-293.
16. Genovart, M, Juste, J, Contreras-Díaz, H & Oro, D (2012) Genetic and phenotypic differentiation between the critically endangered Balearic shearwater and neighboring colonies of its sibling species. J Her 103: 330-341.
17. Austin, JJ, Bretagnolle, V & Pasquet, E (2004) A global molecular phylogeny of the small *Puffinus* shearwaters and implications for systematics of the little-Audubon’s shearwater complex. Auk 121: 847-864.
18. Kuroda, N (1967) Note on the whitish underparts of *Puffinus tenuirostris* and a supposed hybrid between *P. griseus*. J Yamashina Inst Ornith 5: 194-197. In [16].
19. Simeone, A, Hiriart-Bertrand, L, Reyes-Arriagada, R, Halpern, M, Dubach, J, Wallace, R, Putz, K & Luthi, B (2009) Heterospecific pairing and hybridization between wild Humboldt and magellanic penguins in southern Chile. Condor 111: 544-550.
20. White, RW & Clausen, AP (2002) Rockhopper *Eudyptes chrysocome chrysocome* x macaroni *E. chrysolophus* penguin hybrids apparently breeding in the Falkland Islands. Mar Ornith 30: 40-42.
21. Woehler, EJ & Gilbert, CA (1990) Hybrid rockhopper-macaroni penguins, interbreeding and mixed species pairs at Heard and Marion Islands. Emu 90: 198-210.
22. Hull, CL & Wiltshire, A (1999) An apparent hybrid royal x rockhopper penguin at Macquarie Island. Aust Bird Watcher 183: 95-100. In [20].
23. Simpson, KNG (1985) A rockhopper x royal penguin hybrid from Macquarie Island. Aust Bird Watcher 11: 35-45. In [21].
24. Napier, RB (1968) Erect-crested and rockhopper penguins interbreeding in the Falkland Islands. *B.A.S. Bulletin* 16: 71-72. In [20].
25. Taylor, SA, Zavalaga, CB & Friesen, VL (2010) Hybridization between blue-footed (*Sula nebouxii*) and Peruvian (*Sula variegata*) boobies in Northern Peru. Waterbirds 33: 251-257.
26. Taylor, SA, Anderson, DJ, Zavalaga, CB & Friesen, VL (2012) Evidence for strong assortative mating, limited gene flow, and strong differentiation across the blue-footed / Peruvian booby hybrid zone in northern Peru. J Avian Biol 43: 311-324.
27. Figueroa, J & Stucchi, M (2008) Possible hybridization between the Peruvian booby *Sula variegata* and the blue-footed booby *S. nebouxii* in Lobos de Afuera Islands, Peru. Mar Ornith 36: 75-76.
28. Ayala, L (2006) Apparent hybridization between blue-footed *Sula nebouxii* and Peruvian *S. variegata* boobies on Lobos de Tierra Island, Peru. Mar Ornith 34: 81-82.
29. Nelson, JB (1978) The Sulidae: gannets and boobies. Oxford University Press. In [27].
30. Pitman, RL & Jehl, JR (1998) Geographic variation and reassessment of species limits in the “Masked” Boobies of the eastern Pacific Ocean. Wilson Bulletin 110: 155-170. In [27].
31. Dearborn, DC & Anders, AD (2000) Occurrence and breeding behaviour of lesser frigatebirds (*Fregata ariel*) on Tern Island, northwestern Hawaiian Islands. Pacific Seabirds 27: 2-5.
32. Bertellotti, M, Donázar, JA, Blanco, G & Forero, G (2003) Imminent extinction of the guanay cormorant on the Atlantic South American coast: a conservation concern? Biodiv & Conser 12: 743-747.
33. Malacalza, VE (1991) External characters in the offspring resulting from cross-breeding between cormorant species. Colonial Waterbirds 14: 180-183.
34. Taylor, SA, Patirana, A, Birt, T & Friesen, V (2012) Cryptic introgression between murre sister species (*Uria* spp.) in the Pacific low Arctic: frequency, cause, and implications. Pol Biol 35: 931-940.
35. Friesen, VL, Barrett, RT, Montevecchi, WA & Davidson, WS (1993) Molecular identification of a backcross between a female common murre x thick-billed murre hybrid and a male common murre. Can J Zool 71: 1474-1477. In [34].
36. Cairns, DK & DeYoung, B (1981) Back-crossing of a common murre (*Uria aalge*) and a common murre-thick-billed murre hybrid (*U. Aalge x U. lomvia*). Auk 98: 847.
37. Tschanz, B & Wehrlin, J (1968) Kreuzung zwischen trottellumme und dickschnabellumme. *Fauna* *(Oslo*) 21: 53-55. In [34].
38. Wilhelm, SI, Walsh, CJ, Stenhouse, IJ & Storey, AE (2001) A possible common guillemot (*Uria aalge*) x razorbill (*Alca torda*) hybrid. Atlantic Seabirds 3: 85-88.
39. Whittam, RM (1998) Interbreeding of roseate and Arctic terns. Wilson Bulletin 110: 65-70.
40. Zingo, JM, Church, CA and Spendelow, JA (1994) Two hybrid common x roseate terns fledge at Falkner Island, Connecticut. Connecticut Warbler 14: 50-55. In [39].
41. Hays, H (1975) Probable common x roseate tern hybrids. Auk 92: 219-234.
42. Robbins, CS (1974) Probable interbreeding of common and roseate terns. British Birds 67: 168-170.
43. Davis, AH (1982) Terns showing mixed characters of black and white-winged black terns. British Birds 75: 579-580. In [39].
44. Vinicombe, K (1980) Tern showing mixed characters of black tern and white-winged tern. British Birds 73: 223-225. In [39].
45. Hill, NP (1965) The Birds of Cape Cod, Massachusetts. William Morrow, New York. In [39].
46. Dies, JI (2001) Bare-part colours of juvenile hybrid lesser crested x Sandwich tern. British Birds 94: 42.
47. Dies, JI & Dies, B (1998) Hybridization between lesser crested and Sandwich tern in Valencia, Spain, and plumage of offspring. British Birds 91: 165-170.
48. Steele, J & McGuigan, C (1989) Plumage features of a hybrid juvenile lesser crested x sandwich tern. Birding World 2: 391-392.
49. Verroken, L (1990) Presumed hybrid sandwich x lesser crested tern. Birding World 3: 418-419. In [39].
50. Collins, CT (1997) Hybridization of a sandwich and elegant tern in California. Western Birds 28: 169-173.
51. Reinhardt, K, Blechschmidt, K, Peter, H-U & Montalti, D (1997) A hitherto unknown hybridization between Chilean and South Polar skua. Pol Biol 17: 114-118.
52. Peter, HU, Reinhardt, K, Blechschmidt, K & Montalti, D (1994) Ecology and systematics of skuas in the hybrid zone. J Ornith 135: 48 (Poster Abstract XXI International Ornithological Congress, Vienna 1994). In [51].
53. Parmalee, DF (1988) The hybrid skua a southern ocean enigma. Wilson Bulletin 100: 345-356. In [51].
54. Pietz, PC (1987) Feeding and nesting ecology of sympatric South Polar and brown skuas. Auk 104: 617-627. In [51].
55. Devillers, P (1978) Distribution and relationships of South American skuas. Gerfault 68: 374-417. In [51].
56. Andersson, M (1999) Hybridization and skua phylogeny. Proc Roy Soc Lon B 266: 1579-1585.
57. Sternkopf, V, Liebers-Helbig, D, Ritz, MS, Zhang, J, Helbig, AJ & de Knijff, P (2010) Introgressive hybridization and the evolutionary history of the herring gull complex revealed by mitochondrial and nuclear DNA. BMC Evol Biol 10: 348.
58. Godfrey, WE (1973) More presumed hybrid gulls: *Larus argentatus* and *L. marinus*. Canadian Field-Naturalist 87: 171-172. In [62].
59. Jehl, JR, Jr. (1960) A probable hybrid of *Larus argentatus* and *L. marinus*. Auk 77: 343-345. In [62].
60. Andrle, RF (1972) Another probable hybrid of *Larus marinus* and *L. argentatus*. Auk 89: 669-671. In [62].
61. Andrle, RF (1973) A second possible hybrid of *Larus marinus* and *L. argentatus*. Canadian Field-Naturalist 87: 170-171. In [62].
62. Pierotti, R (1987) Isolating mechanisms in seabirds. Evol 41: 559-570.
63. Pálsson, S, Vigfúsdóttir, F & Ingólfsson, A (2009) Morphological and genetic patterns of hybridization of herring gulls (*Larus argentatus*) and glaucous gulls (*L. hyperboreus*) in Iceland. Auk 126: 376-382.
64. Vigfúsdóttir, F, Pálsson, S, & Ingólfsson, A (2008) Hybridization of glaucous gull (Larus hyperboreus and herring gull (Larus argentatus) in Iceland: mitochondrial and microsatellite data. Phil Trans Roy Soc Lon B 363: 2851-2860.
65. Spear, LB (1987) Hybridization of glaucous and herring gulls at the Mackenzie Delta, Canada. Auk 104: 123-125.
66. Ingólfsson, A (1970) Hybridization of glaucous gulls *Larus hyperboreus* and herring gulls *L. argentatus* in Iceland. Ibis 112: 340-362.
67. Bell, DA (1996) Genetic differentiation, geographic variation and hybridization in gulls of the *Larus glaucescens-occidentalis* complex. Condor 98: 527-546.
68. Bell, DA (1997) Hybridization and reproductive performance in gulls of the *Larus glaucescens-occidentalis* complex. Condor 99: 585-594.
69. Yésou, P (1991) The sympatric breeding of *Larus fuscus, L. cachinnans* and *L. argentatus* in western France. Ibis 133: 256-263.
70. Harris, MP, Morley, C & Green, GH (1978) Hybridization of herring and lesser black-backed gulls in Britain. Bird Study 25: 161-166. In [62].
71. Gay, L, Neubauer, G, Zagalska-Neubauer, M, Debain, C, Pons, J-M, David, P & Crochet, P-A (2007) Molecular and morphological patterns of introgression between two large white-headed gull species in a zone of recent secondary contact. Mol Ecol 16: 3215-3227.
72. Panov, EN & Monzikov, DG (1999) Intergradation between the herring gull *Larus argentatus* and the southern herring gull *Larus cachinnans* in European Russia. Russ J Zool 3: 129-141.
73. Williamson, FSL & Peyton, LJ (1963) Interbreeding of glaucous-winged and herring gulls in the Cook Inlet region, Alaska. Condor 65: 24-28.
74. Strang, CA (1977) Variation and distribution of glaucous gulls in western Alaska. Condor 79: 170-179. In [67].
75. Firsova, LW & Levada, AV (1982) Ornithological finds at the south of Koriak plateau. *Ornithologia* 17: 112-118 (in Russian). In [67].
76. Gurr, L (1967) Interbreeding of *Larus novaehollandiae scopulinus* and *Larus bulleri* in the wild in New Zealand. Ibis 109: 552-555.
77. Taverner, JH (1970) Mediterranean gulls nesting in Hampshire. British Birds 63: 67-79.
78. Voous, KH (1960) Atlas of European Birds. London, Nelson. In [76].
79. Sinclair, JC (1977) Interbreeding of grey-headed and Hartlaub’s gulls. Bokmakierie 29: 70-71.
